# Epidemiology and antimicrobial resistance profile of invasive non-typhoidal *Salmonella* from the Philippines Antimicrobial Resistance Surveillance Program, 2014–2018

**DOI:** 10.5365/wpsar.2023.14.1030

**Published:** 2023-09-06

**Authors:** Sonia B Sia, Ferissa B Ablola, Marietta L Lagrada, Agnettah M Olorosa, June M Gayeta, Marilyn T Limas, Manuel C Jamoralin, Polle Krystle V Macaranas, Holly Grace O Espiritu, June Janice B Borlasa, Emmanuel Alfred S Villamin, Ma Cecilia G Alea, Janine Elizabeth V Guia

**Affiliations:** a Research Institute for Tropical Medicine, Department of Health, Manila, Philippines.; * These authors contributed equally.

## Abstract

**Objective:**

The epidemiology of invasive non-typhoidal *Salmonella* (iNTS) in the Philippines is not well elaborated. The present study describes the serotype distribution and antimicrobial susceptibility patterns of iNTS in the Philippines from 2014 to 2018.

**Methods:**

Invasive NTS isolates were collected through the Department of Health’s Antimicrobial Resistance Surveillance Program (ARSP). The identification of the isolates was confirmed using automated (Vitek®, bioMérieux, Marcy l’Étoile, France) and conventional methods. The isolates were serotyped using the slide agglutination method, and susceptibility testing was performed using Clinical and Laboratory Standards Institute guidelines. Demographic data were collected from the ARSP database.

**Results:**

There were 138 isolates collected from human invasive specimens with 97.8% from blood samples. The most common serotypes were *Salmonella* Enteritidis (*n* = 84, 60.9%) and *Salmonella* Typhimurium (*n* = 18, 13.0%). Most of the isolates were from males (*n* = 88, 63.8%) and from the 0–5-year age group (*n* = 61, 44.2%). The proportions of iNTS isolates resistant to first-line antibiotics were as follows: ampicillin (23.2%), chloramphenicol (9.6%), ciprofloxacin (8.7%), ceftriaxone (2.2%) and trimethoprim-sulfamethoxazole (8.8%). The proportion of isolates with multidrug resistance was 13.0% (18/138) with the most common resistance profile being resistance to ampicillin-chloramphenicol-ciprofloxacin from *Salmonella* Enteritidis isolates (*n* = 5).

**Discussion:**

Resistance to first-line antibiotics limits the therapeutic choices for *Salmonella* infection. Relevant local antimicrobial resistance data on iNTS may support appropriate empiric therapy among vulnerable populations.

*Salmonella enterica* causes a wide range of infections among humans. Of its six subspecies, *S. enterica* subspecies enterica was solely associated with diseases among warm-blooded animals. (*1*) Only a small subset of serovars included in this subspecies can cause systemic infection-like typhoidal illnesses (*S*. Typhi and *S*. Paratyphi serovars). (*2*) However, the majority of this subspecies can commonly induce self-limiting diarrhoea, which is referred to as non-typhoidal *Salmonella* (NTS) gastroenteritis. (*3*) Invasive (bloodstream and extra-intestinal) NTS (iNTS) was also observed among persons living with HIV and immunocompromised children. (*4*) *S. enterica* serovars Typhimurium and Enteritidis were the two most common NTS associated with systemic infections that show features of typhoid fever. Globally, there were over 2.1 million cases and 416 000 deaths per year from iNTS infections, with a case fatality rate of more than 20% in children even with the suggested treatment. (*5*)

The epidemiology and antimicrobial resistance pattern of iNTS in Asia is not well documented, with limited reports from India, China, Taiwan (China) and Thailand. (*6*) There are no local data on serotype distribution and susceptibility profile of iNTS in the Philippines. Antimicrobial resistance data on iNTS isolates are of paramount importance as antibiotic treatment of infections due to these isolates is necessary. (*7*) NTS gastroenteritis is usually non-fatal to immunocompetent individuals; however, invasive infection due to NTS could be fatal to immunocompromised populations such as those having malnutrition and people living with HIV. (*8*) With 21.5% of children < 5 years old in the Philippines being underweight (*9*) and with the sustained rapid increase in new HIV infections in the country, relevant local antimicrobial resistance (AMR) data on iNTS may support appropriate empiric therapy among vulnerable populations. (*10*)

This study describes the epidemiology of NTS serotypes causing invasive infections and the antimicrobial resistance patterns of these isolates in the Philippines from 2014 to 2018.

## Methods

### Study setting and population

The Philippines Department of Health (DOH) Antimicrobial Resistance Surveillance Program (ARSP) is a sentinel laboratory-based surveillance system of antimicrobial-resistant aerobic bacteria detected from clinical specimens. Culture and antimicrobial susceptibility data are collected from 24 tertiary hospitals located in 16 regions of the Philippines. There are eight sentinel sites in the National Capital Region and one or two sentinel sites in each of the other regions. All sentinel sites implement standard methods for culture and susceptibility testing based on the WHO manual for the laboratory identification and antimicrobial susceptibility testing of bacterial pathogens of public health importance in the developing world (*11*) and updated Clinical Laboratory Standards Institute (CLSI) references for antibiotic susceptibility testing and quality control. (*12*, *13*) The sentinel sites participate in an external quality assessment scheme conducted by the reference laboratory to ensure the quality of laboratory results. Staff from the Antimicrobial Resistance Surveillance Reference Laboratory (ARSRL) conduct periodic monitoring visits to sentinel sites to ensure that laboratory protocols are consistently being observed.

### Data collection

Microbiological and demographic data from sentinel sites were entered into WHONET, a database designed for the management and analysis of microbiology laboratory data focusing on the analysis of antimicrobial susceptibility test results. A data extraction tool was used by ARSP to collect data from the WHONET database. Information on the age, sex, sentinel site, specimen type and initial serotyping result was collected for each isolate.

Isolates included in this study were positive for NTS. They were isolated either alone or in combination with another pathogen from blood, cerebrospinal fluid, tissue, fluid or respiratory specimens from January 2014 to December 2018. Only the first isolate from patients with multiple positive blood cultures for the same NTS serogroup and antimicrobial susceptibility profile was included in the study.

### Microbiological procedures

All isolates received by the ARSRL were confirmed using both automated (Vitek®, bioMérieux, Marcy l’Étoile, France) and conventional methods at the reference laboratory. The isolates were serotyped using the Sven Gard method for slide agglutination using Denka Seiken antisera (Tokyo, Japan) and S&A Reagents serotest (Bangkok, Thailand). The antigenic formulae obtained were classified according to the White–Kauffmann–Le Minor scheme, as recommended by the WHO Collaborating Centre for Reference and Research on *Salmonella*. (*2*)

Antimicrobial susceptibility testing for ampicillin, ceftriaxone, chloramphenicol, ciprofloxacin and trimethoprim-sulfamethoxazole was performed using both automated (Vitek®) and conventional methods (Kirby Bauer disk diffusion and gradient diffusion method). Antimicrobial susceptibility results were interpreted using CLSI interpretive criteria (M100Ed28E). The proportion of the isolates that were resistant was generated using WHONET 5.6 software with only the first isolate per calendar year included. Quality control analyses for iNTS serotyping and antimicrobial susceptibility testing were conducted using *Escherichia coli* ATCC 25922.

## Results

There were 138 isolates collected from ARSP from 2014 to 2018. Among the isolates, 130 were characterized to serotypes and eight were assigned to serogroups. The most common serotypes were *Salmonella* Enteritidis (*n* = 84, 60.9%) and *Salmonella* Typhimurium (*n* = 18, 13.0%; **Table 1**).

**Table 1:** Frequency of invasive non-typhoidal *Salmonella* isolates from the Antimicrobial Resistance Surveillance Program, the Philippines, 2014–2018 (*n* = 138)

Salmonella serotype/ serogroup	2014	2015	2016	2017	2018	TOTAL
Enteritidis	4	14	13	26	27	84
Typhimurium	4	1	6	3	4	18
Virchow	2	–	1	1	1	5
Group C	–	–	1	–	3	4
Choleraesuis var. Kunz	1	1	1	–	–	3
Group B	1	–	–	2	–	3
Anatum	–	–	–	1	1	2
Kentucky	–	–	–	1	1	2
Stanley	–	1	–	–	1	2
Aberdeen	–	1	–	–	–	1
Ajiobo	–	–	–	–	1	1
Choleraesuis	–	1	–	–	–	1
Derby	–	–	–	–	1	1
Eastbourne	–	1	–	–	–	1
Emek	–	1	–	–	–	1
Group A	–	–	–	1	–	1
Heidelberg	–	–	1	–	–	1
Hillingdon	–	–	1	–	–	1
Javiana	1	–	–	–	–	1
Nessziona	–	–	–	–	1	1
Ohio	–	–	1	–	–	1
Rissen	1	–	–	–	–	1
Saintpaul	–	–	–	–	1	1
Tallahassee	–	–	–	1	–	1
**TOTAL**	**14**	**21**	**25**	**36**	**42**	**138**

**Fig. 1:**
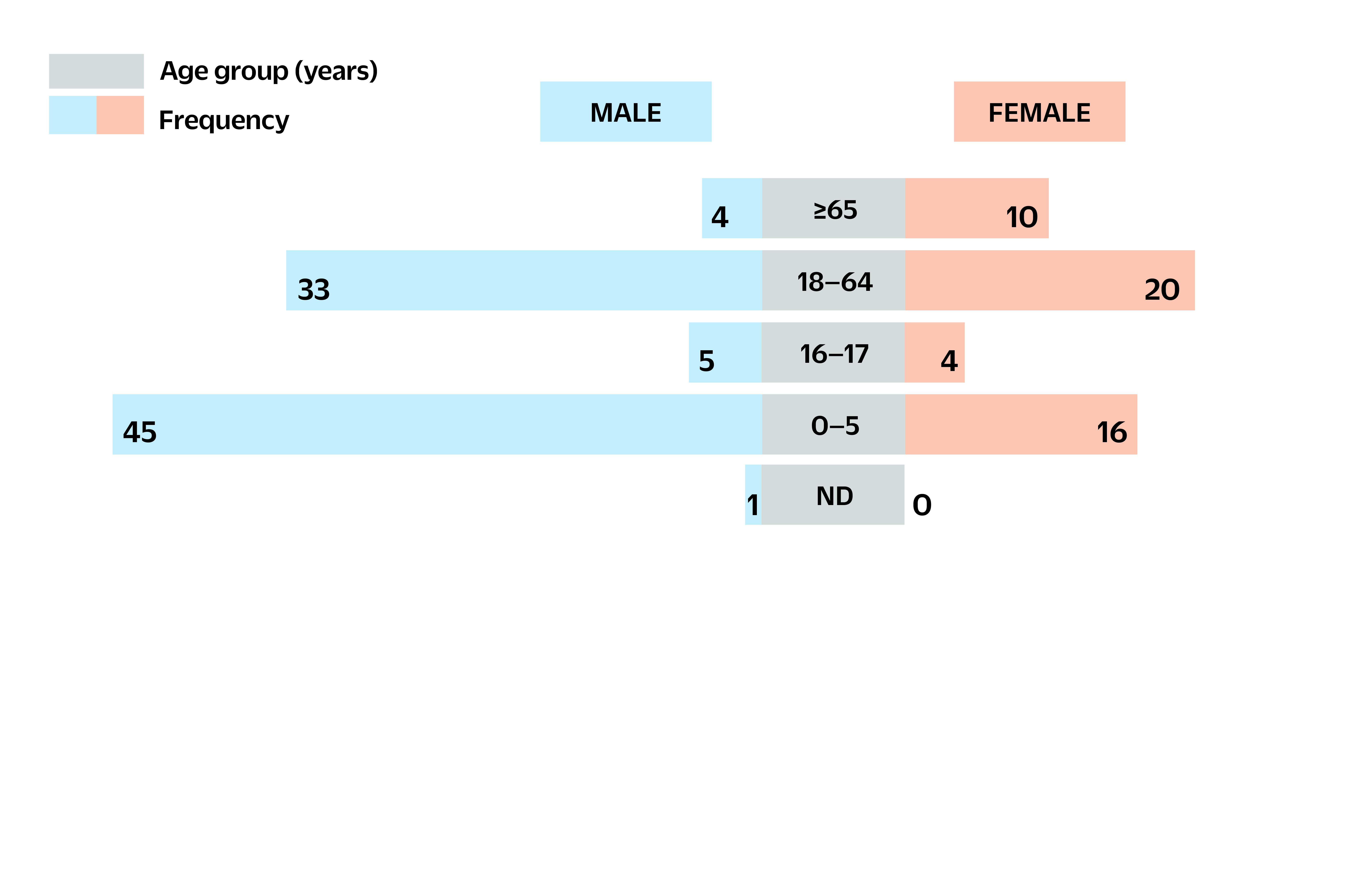
Frequency of invasive non-typhoidal Salmonella isolates by age group and sex, Antimicrobial Resistance Surveillance Program, the Philippines, 2014–2018

The majority of isolates were from blood samples (*n* = 135, 97.8%), while three were from cerebrospinal fluid (2.2%). Most of the isolates were collected from Luzon (*n* = 63, 46.4%) (**Table 2**). Invasive NTS isolates were more common in males (*n* = 88, 63.8%) than females (*n* = 50, 36.2%). The 0–5-year age group (*n* = 61, 44.2%) had the highest proportion of iNTS isolates (**Fig. 1**), with most paediatric patients infected with *Salmonella* Enteritidis. The number of isolates increased annually, with the highest number collected in 2018 (**Table 1**).

[**insert** Figure 1]

**Table 2:** Invasive non-typhoidal *Salmonella* serotypes per region from the Antimicrobial Resistance Surveillance Program, the Philippines, 2014–2018

*Salmonella* serotype	Region			
	Luzon^a^	Visayas^b^	Mindanao^c^	National Capital Region
Enteritidis	41	17	12	14
Typhimurium	10	2	5	1
Virchow	2	–	2	1
Group C	2	2	–	–
Choleraesuis var. Kunz	2	–	–	1
Group B	2	1	–	–
Anatum	1	1	–	–
Kentucky	–	2	–	–
Stanley	1	–	1	–
Other	2	10	–	2
**TOTAL**	**63**	**35**	**20**	**19**

^a^ Luzon contains seven sentinel sites.

^b^ Visayas contains four sentinel sites.

^c^ Mindanao contains five sentinel sites.

**Fig. 2:**
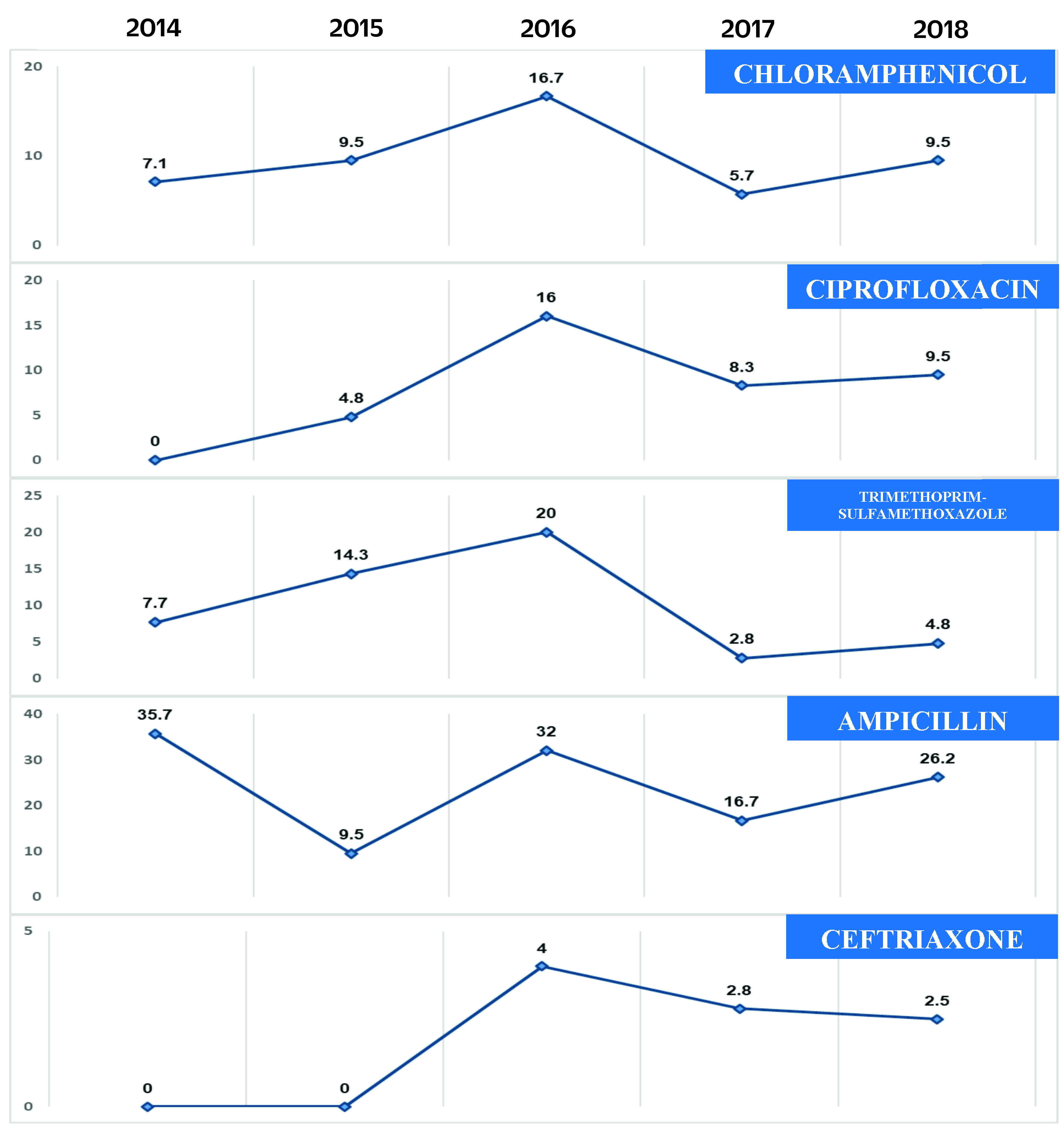
Annual antimicrobial resistance rates of invasive non-typhoidal Salmonella isolates, Antimicrobial Resistance Surveillance Program, the Philippines, 2014–2018

The proportion of isolates resistant to antibiotics over the 5-year study period was less than 10% for each antibiotic, except for ampicillin, which was 23.2% (**Table 3**). Resistance to ciprofloxacin and ceftriaxone was present in 2015 and 2016 and persisted in 2018. The proportion of isolates resistant to chloramphenicol, ciprofloxacin, trimethoprim-sulfamethoxazole, ampicillin and ceftriaxone was highest in 2016, with a subsequent decrease of resistance in 2017 (**Fig. 2**). The observed decreases in 2017 were all statistically significant. In 2018, the proportion of isolates resistant to all antibiotics increased except for ceftriaxone; however, these increases were not significant (**Fig. 2**).

[**insert** Figure 2]

**Table 3:** Cumulative proportion of invasive non-typhoidal *Salmonella* isolates that were resistant by antibiotic, Antimicrobial Resistance Surveillance Program, the Philippines, 2014–2018

Antibiotic	% resistant	95% confidence interval
Ampicillin	23.2	16.6–31.3
Cefotaxime	1.4	0.2–5.6
Ceftriaxone	2.2	0.6–6.8
Chloramphenicol	9.6	5.4–16.2
Ciprofloxacin	8.7	4.8–15.0
Trimethoprim- sulfamethoxazole	8.8	4.8–15.2

There were 18 multidrug-resistant (MDR) isolates (**Table 4**), that is, they were resistant to at least three antibiotic classes, giving an overall MDR proportion of 13.0%. The most common MDR resistance profile was resistance to ampicillin-chloramphenicol-ciprofloxacin, and these isolates were all *Salmonella* Enteritidis (*n* = 5 isolates).

**Table 4:** Multidrug-resistant^a^ invasive non-typhoidal *Salmonella* isolates, Antimicrobial Resistance Surveillance Program, the Philippines, 2014–2018

Resistance profile	Frequency
AMP-CHL-CIP	5
AMP-CIP-SXT	3
AMP-CHL-CIP-SXT	3
CHL-CIP-SXT	2
AMP-CHL-SXT	2
CRO-CHL-CIP	1
AMP-CRO-CTX-CHL-CIP	1
AMP-CRO-CTX-CIP-SXT	1
**Total**	**18**

^a^ Resistant to three or more drug classes.

AMP: ampicillin; CHL: chloramphenicol; CIP: ciprofloxacin; CRO: ceftriaxone; CTX: cefotaxime; SXT: trimethoprim-sulfamethoxazole.

## Discussion

The most common iNTS identified from the ARSP between 2014 and 2018 were *Salmonella* Enteritidis and *Salmonella* Typhimurium. This finding is similar to that identified in the Typhoid Fever Surveillance in Africa Program in 2010–2014, (*14*) the Hospital for Tropical Diseases in Viet Nam in 2008–2013, (*15*) from 461 iNTS isolates collected in India in 2010–2020, (*16*) and from genome sequencing-confirmed invasive *Salmonella* isolates collected from tertiary hospitals in the Nigeria Antimicrobial Surveillance Network. (*17*)

More adult males were affected by iNTS infections compared with adult females. Although such distribution may be attributed to male behavioural factors such as higher risks in food handling, preparation and consumption, (*18*) it could likewise be a factor of the prevailing sex ratio in the country, as there were more males than females in the Philippines from 2014 to 2018. (*19*) Most iNTS isolates were from patients aged 0–5 years. Underdeveloped immune systems, malnutrition and presence of comorbidities may predispose this age group to iNTS infections. (*3*, *20*)

Invasive NTS infections were higher in Luzon, which has both rural and urban areas. Luzon is the largest and most populous island in the Philippines, which may be the reason for the higher number of reported cases from this island group in the study. Cruz Espinoza et al. concluded in their study that salmonellosis is mainly a disease of high-density population, (*20*) and in Luzon, there are urban in-migration generating slum areas with poor water access and poor hygiene practices (*21*) that may increase the risk of food and waterborne diseases among residents. Having 15 of the 24 sentinel sites of the ARSP located in Luzon likely contributed to the preponderance of iNTS infections from Luzon. Variations in the diagnostic practices of physicians and capacities of the laboratories in the different sentinel sites may have also contributed to differences in the number of iNTS in the different island groups shown in this study.

The proportion of iNTS isolates resistant to first-line antibiotics (chloramphenicol, ampicillin, trimethoprim-sulfamethoxazole, ceftriaxone) remained below 10%, except for ampicillin at 23%. This is relatively low compared with neighbouring Thailand, which has reported resistance to ampicillin as high as 68.2%. (*6*) Among iNTS isolates from children in China, Taiwan (China), resistance to ceftriaxone and ciprofloxacin was noted to be 5.6% and 30.6%, respectively. (*22*) That there was resistance to the locally recommended empiric treatment of severe NTS infections of ciprofloxacin, a fluoroquinolone, and ceftriaxone, a third-generation cephalosporin, (*23*, *24*) is a concern.

Among the iNTS isolates in this study, MDR was relatively low, with the most common resistance profile being to ampicillin, chloramphenicol and ciprofloxacin among *Salmonella* Enteritidis isolates. Low local MDR rates may allow for wider empiric treatment selection for iNTS infections. Continued emergence of resistance to these antibiotics may further limit treatment options for iNTS.

The isolates in this study were from regional hospitals that are sentinel sites for the ARSP. These hospitals cater to patients from towns and cities within the hospital vicinity and may not be representative of all hospital patients in the Philippines. There may be resistance variations in local areas not represented in programme data. Given that the ARSP data are from routine clinical samples, and not all patients have samples taken, there may be differences in isolates selected for microbiological culture, which may also introduce bias in the resistance data presented. In addition, the small number of isolates included in this study is a limitation, as the performance of culture and susceptibility tests in the sentinel sites is dependent on the diagnostic habits of the clinicians.

### Conclusion

To our knowledge, this study is the first extensive report of iNTS for the Philippines, which showed that in 2014–2018, the most common serotypes among iNTS in the Philippines were *Salmonella* Enteritidis and *Salmonella* Typhimurium. Continued surveillance of AMR among iNTS may support appropriate empiric therapy among vulnerable populations and can contribute to the reduction of the selection and spread of resistant infections. Genomic epidemiology of resistant iNTS may lead to a better understanding of transmission patterns and emergence of resistance among these bacteria and may inform varied control measures including vaccine development.
